# An interactive course to enhance self-efficacy of family practitioners to treat obesity

**DOI:** 10.1186/1472-6920-5-4

**Published:** 2005-01-29

**Authors:** Sara Katz, Amiel Feigenbaum, Shmuel Pasternak, Shlomo Vinker

**Affiliations:** 1 Technion – Israel Institute of Technology – Faculty of Medicine, Haifa, Israel; 2 Department of Family Medicine, Sackler School of Medicine, Tel Aviv University, Israel; 3 General Health Services, Holon, Israel

## Abstract

**Background:**

Physicians' awareness of their important role in defusing the obesity epidemic has increased. However, the number of family practitioners who treat obesity problems continues to be low. Self-efficacy refers to the belief in one's ability to organize and execute the courses of action required to produce given attainments. Thus, practitioners who judge themselves incapable of managing obesity do not even try. We hypothesized that practitioners' self-efficacy and motivation would be enhanced as a result of participating in an interactive course designed to enrich their knowledge of obesity management.

**Methods:**

Twenty-nine family practitioners participated in the course, which was accompanied by qualitative interviews. The difference between the physicians' pre-course and post-course appraisals was tested by paired *t*-test. The interviews were analyzed by qualitative methods.

**Results:**

Post-course efficacy appraisals were significantly higher than pre-course appraisals (*p *< 0.0005). A deeper insight on the practitioners' self-efficacy processes was gained through reflection of the practitioners on their self-efficacy during the interviews.

**Conclusions:**

Up-to-date information and workshops where skills, attitudes and social support were addressed were important in making the program effective.

## Background

Obesity is becoming increasingly common and is recognized as a major public health problem worldwide [1-3]. In the UK, the overweight and obese population increased by almost 15% between 1980 and 1992. Similar increases have been noted in many countries, including the USA [4], Sweden [5] and the Netherlands [6]. In Israel, 55% of females and 58% of males aged 25–64 were reported to be overweight or obese (Body Mass Index >25 kg/m^2^) in a Mabat survey [7] in 2000.

Guidelines published in 1996 for the management of obesity recommended setting a modest weight loss and weight maintenance, rather than a targeted ideal weight, as goals [8]. Lately, pharmacological therapies have been proposed as adjuncts to diet and lifestyle changes to improve long-term weight loss [9]. The World Health Organization declared obesity an epidemic in developed countries. Awareness of physicians of their important role in defusing the obesity epidemic has increased. Yet, the number of family practitioners (FP) treating obesity problems continues to be low [10].

Many chronic health problems are exacerbated by unhealthy behaviors and harmful environmental conditions. From the psychological perspective, healthful lifestyles and environmental conditions may yield large health benefits. The widespread adoption of a healthier lifestyle rather than medical technologies has resulted in a substantial decline in premature mortality and morbidity [11]. The media plays a major role in informing the public about health risks. Efforts to convince people to adopt health practices that prevent disease have relied heavily on persuasive communications in health education campaigns [11]. Health benefits are accelerated by community-wide efforts to reduce habits that impair health [12].

Self-efficacy refers to the belief in one's ability to organize and execute the courses of action required to produce a given attainment [11]. Such beliefs influence the courses of action people choose to pursue, how much effort they put into a given endeavor and how long they will persevere in the face of obstacles and failure. If people believe they have no power to produce results, they will not attempt to effect changes. Self-efficacy is not a fixed ability that one has or lacks in one's behavioral repertoire. Rather, it is a thinking process, a generative capability in which cognitive, social, emotional and behavioral sub-skills are organized and effectively orchestrated to serve innumerable purposes. Self-efficacy is concerned not with the number of skills one has, but with what one believes one can do with the skills under a variety of circumstances. Efficacy beliefs operate as a key factor in a generative system of human competence [11,13-15]. Self-efficacy is an important contributor to performance accomplishments, whatever the underlying skills might be [11]. The greater a person's efficacy beliefs, the greater the academic challenges one sets for oneself and the greater their intrinsic interest [16]. Personal efficacy beliefs influence the level of interest in occupational pursuits even when the influence of ability is removed [17,18]. A sense of personal efficacy is constructed through a complex process of self-persuasion. Efficacy beliefs are the product of cognitive processing of diverse sources of information conveyed inactively, vicariously, socially and physiologically. Once formed, efficacy beliefs contribute to the quality of human functioning [11].

Self-efficacy is measured by the strength of a subject's beliefs in the ability to execute requisite activities. Social cognitive theory distinguishes among three basic processes of personal change: the adoption, general usage and maintenance over time of new behavioral patterns [19]. Efficacy beliefs affect each of these phases [11]. Self-efficacy conceptualizes a person's perceived ability to perform on a task as a mediator of performance on future tasks [11,13]. A change in the level of self-efficacy can predict a lasting change in patients' behavior if there are adequate incentives and skills [13-15]. People's beliefs that they can motivate themselves and regulate their own behavior play a crucial role in whether they even consider acting. Thus, practitioners who judge themselves incapable of treating obesity do not even try. Obesity has not been considered a disease till recent years. Physicians did not treat obesity unless they were asked to do so by patients.

To our knowledge and after a literature search, no intervention studies have yet been conducted to change the self-efficacy of FPs in Israel towards treating obese people. Among other courses for which physicians usually receive credits towards their annual professional training, a course was offered by the Israeli Academic Medical Council. The course was initiated by the Israeli Association of FPs and recommended by the Medical Professional Journal of FPs in Israel. Registration in the course was open to all FPs. The present research was a preliminary study. This being the case, the first group of FPs who attended the new course participated in it. Though that group contained a small number of subjects, the importance of investigating a new program contribution to FPs self-efficacy for future research was evident.

The objectives of the course were to enrich the knowledge of FPs with up-to-date information on obesity and to raise their motivation to treat it. The study objective was to determine if an interactive course would raise the self-efficacy of FPs to treat obesity. It was hypothesized that the self-efficacy of FPs would be enhanced as a result of participating in an interactive obesity-treatment course.

## Methods

### Subjects

Twenty-nine FPs (62% female) chose to participate in the course along with other Continuing Medical Education (CME) courses. All participants work as FPs in public health care clinics throughout the country.

### Design

This study was based on a one group, pre-course – post-course test design, without a control group. It was accompanied by qualitative interviews to validate the results of the data analysis.

### Research tools

The strength of the efficacy beliefs of the FPs to treat obesity was estimated by using a five point scale Likert type questionnaire containing nine items (Table 1). The subjects were asked how confident they were in the ability to treat obesity across problem situations. The levels of confidence were as follows: 1. not at all confident, 2. somewhat confident, 3. moderately confident, 4. very confident, 5. completely confident. The questionnaire was filled out before and after the course. The items were generated from the criteria on the domain map that was constructed on the basis of theoretic analyses of knowledge accumulated in the domains of self-efficacy and health behavior change [11,13-15] and consultation with experts. The researchers used the analytic induction method for the theoretic analysis of knowledge. The map's main criteria were: obesity – a serious disease; up-to-date clinical and behavioral knowledge; processes of change, e.g: decision making, planning, monitoring and behavior controlling; resilience; lack of time and social involvement.

**Table 1:** Original domain map criteria and the sequence of criteria in the questionnaire regarding the self-efficacy beliefs of family practitioners to manage obesity

Original sequence of criteria	Sequence in the questionnaire
Efficacy to treat a problem of high priority	1
Efficacy to give up-to-date and correct information	7
Efficacy to persuade, support and help patients make decisions	3
Efficacy to make patient plan behaviors and situations	6
Efficacy to make patient monitor his behavior	4
Efficacy to make patient control behaviors and situations	9
Efficacy to treat obesity regardless of previous failure or unsuccessful experiences	2
Efficacy to treat obesity regardless of lack of time	5
Efficacy to bring about involvement of other people in the patient's behavior change process	8

The Alpha Cronbach for the reliability of the tests was α = 0.88 for the pre-course test and α = 0.90 for the post-course test (Table 2).

**Table 2:** Pre-course (α = 0.88) and post-course (α = 0.90) scale mean, SD, item total correlation, and α if item deleted (n = 29)

Item no.	Mean	SD	Item total correlation	α, if item deleted
Pre-course				
1	0.77	0.13	44.03	0.88
2	0.61	0.15	43.62	0.87
3	0.53	0.16	43.40	0.86
4	0.59	0.16	42.83	0.86
5	0.74	0.15	39.85	0.87
6	0.84	0.15	41.39	0.87
7	0.44	0.13	46.61	0.88
8	0.77	0.16	39.89	0.86
9	0.72	0.14	39.36	0.85
Post-course				
1	0.67	0.16	20.10	0.88
2	0.75	0.16	20.67	0.88
3	0.67	0.16	20.11	0.88
4	0.46	0.14	21.17	0.90
5	0.59	0.15	19.82	0.90
6	0.72	0.15	20.29	0.88
7	0.68	0.15	21.50	0.89
8	0.71	0.16	19.21	0.88
9	0.81	0.16	19.99	0.88

The following aspects of structure validity were examined: (a) for the content aspect, the items matched the domain concept map. Final version was rewritten on the basis of experts' and researchers' comments. The items were finally presented in an nonsequential order to make subjects think while reading the questions and not relate to earlier answers; and (b) for the substantive aspect, FPs were interviewed regarding their self-efficacy to assure that the questions were clear and did not require rewording. The interview was a 30 min. open interview. A physician was given open questions such as: "Describe your feelings and thoughts of efficacy to treat obesity" or "How the self-control lectured help your efficacy perceptions". The subject was free to speak openly on the issue. The interview was actually a verbal reflection of thoughts and feelings of the subjects. It was recorded and later on transcribed by the researchers. The interview was analyzed by the constant comparative qualitative method of analysis [20,21]. Every sentence said by the subject in the interview was considered a unit to be taken into account for the content analysis.

The present study design used paired *t*-test in order to compare pre and post strength of efficacy beliefs for treating obesity.

### Procedure

Participants answered the questionnaire before the start of the course and at the end of the last session, and a few days later participated in an open interview. The course was interactive and contained 12 clinical and psychological lectures given by experts in all subjects relating to obesity (Table 3). Every lecture was followed by a workshop. The program consisted of six monthly sessions. Each session (from 17:00 to 21:00) started with two medical lectures followed by a 90 min workshop, and ended with a panel discussion. In the first workshop FPs said they did not address the problem of obesity unless they were asked to do so by patients. Even if they wanted to relate to it they would feel uncomfortable with it, as if that was none of their business. They reported between 1–3 obesity treatments a day.

**Table 3:** Table of contents of the interactive course: "Monitoring and treating obesity by the family practitioner"

1	Obesity – the epidemiological perspective
2	The pathogenesis and metabolic factors of obesity
3	The clinical approach of the family physician to the obese patient
4	Nutrition, diet and treatment of overweight
5	Self-control and behavior modification – diagnosing and managing the stages of change: the trans-theoretical model, the self-regulation model
6	Physical activity and exercise, lifestyle and energy expenditure
7	Drug treatment of obesity
8	Surgical treatment of obesity
9	The metabolic syndrome
10	The mechanism of hypertension in obesity
11	The diabetic obese patient
12	Infertility of obese women

FPs filled in clinical report forms and presented the cases they treated and tools they used. The cases were discussed during the course and feedback was given by colleagues and experts. The course supplied the FPs with knowledge, skills and psychological tools such as: food and physical activity diaries, decision making tools, self-evaluation tools, self-report tools, self-monitoring tools, persuading tools, stimulus control tools and counter-conditioning tools, to treat obesity.

Medical knowledge and skills were not examined. The course attempted to raise the self-efficacy of FPs by creating a supportive atmosphere, providing feedback, recalling successful experiences, learning from models, bringing patients into the workshops, discussing sociological and psychological problems and allowing reflection of thoughts and emotions. Studies have shown that reflection enhances metacognitive processes such as: self-monitoring, self-evaluation, self-reaction and attribution [11,22-25]. Since self-appraisal of efficacy is a form of metacognition and efficacy beliefs are structured by experience and reflective thoughts, we viewed reflection on obesity issues and on FPs self-efficacy as a forethought process so that the mental process the FPs went through had an effect on the processing of their efficacy appraisals. We expected their appraisals to go through a change [13-15].

## Results

The differences between FP pre and post course efficacy appraisals was tested by paired *t*-test (Table 4) and significant differences were found (Effect size .317).

**Table 4:** Difference between pre and post course efficacy beliefs to treat obesity

Item no.	Mean pre-course	Mean post-course	*t*	df	N
1	3.93	4.48			
2	3.86	4.48			
3	4.21	4.41			
4	3.48	4.17			
5	3.00	3.79			
6	3.34	4.07			
7	4.17	4.55			
8	3.31	4.31			
9	3.41	4.10			
			-3.606*	28	29

*(significant 1-tailed) *p *< 0.0005

All subjects reported enhanced efficacy beliefs to treat obesity. Negative feelings towards the course were not said or written. Yet, efficacy belief enhancement of the group differed among the items (see Table 4). Three FPs left the course right after the first meeting claiming a lack of time in learning and dealing with new perceptions and no time to treat the family holistically. They also said that the time slot of the course was inconvenient.

Some FPs presented cases which had failed to bring about a change in the patient's behavior. Those cases were discussed and the FPs were given strategies and tips for further treatment.

In addition, the interviews resulted in reflection by the FPs on the process of their self-efficacy. Coded sentence units of the interviews were constantly compared while evidence accumulated. Nine main criteria were generated through that systematic analysis. These criteria matched the questionnaire items.

The four criteria that received the most evidence formed four core constructs. The first was the contribution of the course to the FPs. FPs appreciated the importance of up-to-date information, the various treatment perspectives, and the skills they acquired. Knowledge was a precondition for change. FPs talked about the practical tools and tips they acquired. This is illustrated in the example: *"It is **his **will and actions and **our **help"*. The second criterion was the efficacy to persuade patients to treat obesity. FPs described how they could persuade patients to treat obesity in a variety of ways. The third criterion was the FP performance reports. FPs presented their clinical report forms and described how they successfully used the new knowledge, skills, tools and tips they acquired in the course to manage obesity in their clinics. The fourth criterion was efficient time management. Time management scored the lowest on the pre-course questionnaire. Whenever the issue was raised during the course, FPs claimed that time was the main obstacle in treating obesity. By the end of the course, a significant change in their perception of time was noted. Prior to the beginning of the course, only 1–3 clinical report forms on obesity treatment were reported by the FPs; at the end of the course, that number increased to 8–37. After having taken the course, obesity problems were not ignored anymore.

Table 5 shows the criteria listed in the questionnaire, and the criteria generated from coded sentence units mentioned during interviews. The criteria derived from the interviews are illustrated by characteristic examples.

**Table 5:** Questionnaire criteria, criteria coming out of interviews, and examples

Questionnaire criteria	Criteria coming out of interviews	Examples from interviews
1. Efficacy to treat a problem of high priority	a. Course contribution: motivation to learn, and to work harder	- *"I used to treat obesity as something that needed cosmetics now I know it's a disease that needs a cure...and it is my job"* - *"I've a lot more to learn...I wish I had more courses like this one...It's very important...I'm ready to work hard...to do a lot more..."*

2. Efficacy to give up to date precise information	b. Course contribution: enriched important knowledge, treatment perspectives, skills, practical tools and tips	- *"Now I have the tools, a lot of information...now I have the right perspective...I shouldn't be angry with him but supportive...with the tools it's much easier...It's **his **will and actions and **our **help. I feel I know how to help him...the course was helpful"*

3. Efficacy to persuade, support and help patients make decisions	c. Efficacy to persuade patients to treat obesity	- *"Today I'm more patient...I know when it's the right moment to bring up the issue...and I know how to do it"* - *"I can use emotions to persuade him"* - *"Before taking the course, I wasn't self-confident enough to do it, now I feel free to talk about this...I can show him statistics on obesity..."*

4. Efficacy to make patients plan behavior and situations	d. Efficacy to make patients plan, monitor and control health behavior	- *"Since he is aware of his expectations I can make him plan or act..."* - *"under my guidance he can see what's right and what's wrong... and he can change things..."* - *"when he understands the process he can initiate behavior"*
5. Efficacy to make patients monitor behavior		
6. Efficacy to make patients control behavior and situations		

7. Efficacy to treat obesity regardless of previous failures or unsuccessful experiences	e. FP performance reports	- *"What you taught in the course works!"* - *"Now that I know that taking small steps is better than expecting a dramatic weight loss – it is easier...and the patient is happier.* *I now treat 10 obese people..."* - *"I now treat 8 people, before taking the course, I did not treat any"* - *"I have 9, I was given feedback by a patient... she said: I lost two pounds, thank **you**!...you are great..."*

8. Efficacy to treat obesity regardless of lack of time.	f. Efficient time management	- *"I'll tell you my secret, everybody starts work at 8.00, I com at 7.00.* *I'm ready to do it, I want to succeed, after all, it is my job"* - *"I make a double appointment for an obese patient..."* - *"If I treat obesity now, I save time, I won't have to treat other diseases in the future"* - *"I keep thinking about how to be more efficient, I have to do something about the time!"* - *"I know that when I want something I find the time"*

9. Efficacy to bring about other people's involvement in the patient's behavior change process	g. Better done with someone's help	- *"I tell her, doing it alone is too difficult. You should bring your husband or a friend to the next visit"*

	h. FP weight loss	- *"I lost weight as a result of the course"*

	i. Enhancing efficacy beliefs by reflection, feedback and supportive climate	- *"Did you hear the FPs' reaction to my presentation?...Wow!... that was great...I understood I had great success"* - *"I was given feedback by patients, now I know I can!"* - *"The best thing that happened to me in this course is that it made me think"* - *"I could open myself to talk about things that I hadn't done before...I think it's because of the warm climate"*

## Discussion

The purpose of the study was to discover whether participation in an interactive course would result in a change in the efficacy beliefs of FPs to treat obesity. Results show a significant difference between pre and post course beliefs. The increase was satisfactory (>4 in a scale of 1–5) and the results of the qualitative analysis indicate that the criteria derived from the interviews matched those of the questionnaire. When speaking openly, FPs addressed the same issues that made up the domain theoretical concept map.

Several researchers have suggested that quantitative efforts in the study of self-efficacy should be complemented by qualitative studies aimed at gaining a deeper understanding of attitudes and emotions [22-24]. The criteria obtained from the interviews not only matched the criteria in the questionnaire but also enriched our knowledge with a deeper understanding of the attitudes and thoughts of the FPs about the course and the way they looked at obesity management after the course. All sentence units of the interviews were taken into account for content analysis.

The study showed that acquisition of knowledge and skills enable a person to meet personal standards of merit that tend to heighten beliefs of personal efficacy [11]. In judging their efficacy, individuals necessarily unite personal agency with means. They act on beliefs of how well they can use prescribed means [10]. The FPs felt the course equipped them with appropriate knowledge and means for treating obese patients. This was expressed in many sentence units. Another important psychological role in all phases of behavior change was the efficacy to persuade patients to treat obesity. The FPs' presentations of clinical report forms and descriptions of success to manage obesity in their clinics support research findings that efficacy beliefs predict both intentions and behavior [10]. The FPs challenged time management as efficiently as they could, which is another important change in the perception of their role as FPs.

The impact of the questions analyzed in this study extends beyond the issues asked by the questionnaire. New insights were gained through qualitative analysis: FPs analyzed the process they experienced during the course. They described how their efficacy beliefs were enhanced through reflection, feedback and the supportive climate of the course. Studies have shown that reflection enhances self-efficacy processes since self-appraisal of efficacy is structured by experience and reflective thought [25]. FPs reported that reflection on thoughts and emotions helped them construct their beliefs. Feedback regarding the quality of one's work progressively raises perceived efficacy, which, in turn, predicts subsequent performance. This is illustrated in studies of self-regulated productivity [26]. The feedback the FPs received from their colleagues and the experts on the quality of their reported experiences enhanced their efficacy beliefs. The FPs feedback on the course showed that the workshops contributed most to their self-efficacy. FPs explained that they had got the chance to "bring the clinic into the workshop", to discuss their performance, to get feedback on it, and to be stimulated to reflect on the treatment and on themselves as physicians. Incentives for mastering activities contribute to the growth of interest and perceived efficacy[10]. The credit points FPs received for their professional training served as an incentive that fostered performance accomplishments. The FPs reported on an increased number of obesity treatments which was an important gain of this educational program. There were no other absences except for the 3 who had left after the first session. At the end of the course FPs requested that the course be continued.

In summary, an effective program of widespread change in health practices includes four major components. The first is informational and intended to increase the physician's awareness and knowledge of the subject. The second involves development of skills needed to translate informed concerns into effective action. The third is aimed at building a robust sense of self-efficacy to support the exercise of control in the face of difficulties that inevitably arise. This is achieved by providing repeated opportunities for guided practice and corrective feedback in applying the skills in simulated situations that people are likely to encounter. The final component involves creating social support for desired changes. The present program contained all these components.

The limitations of the study were the small number of participants and the reliance on one motivated group of FPs. These limitations result from the fact that this was the first course organized by the Israeli Association of Family Physicians on the subject of obesity. It was important to study the effect of the program on FPs self-efficacy for future continuing education and research.

We recommend studying the effect of interactive courses on the lifestyle and weight loss of FPs. Future research should consider randomized samples from larger courses and analyses of the correlation between course success and actual FPs performance, FPs' self-efficacy and treatment outcomes e.g: BMI change, Lipids and BP. We also recommend studying the differences in obesity treatment outcomes and in general health feelings between patients whose FPs attended obesity courses and patients whose FPs did not.

Practical recommendations for Continuing Medical Education planners would be to focus on workshops rather than on lectures, to enhance those processes the FPs felt less efficacious to go through and to have guidance of Endocrinology experts' as well as Psychology and Education specialists to improve communication with patients and their families in an effort to enhance motivation to loose weight. It is also recommended to bring patients to the workshops to reflect on the treatment they have received.

## Competing interests

The author(s} declare that they have no competing interests.

## Authors' contributions

SK and AF conceived and designed the study, participated in the collection, analysis and interpretation of data and drafted the manuscript. SV Participated in the statistical analysis, interpretation of data and draft of the manuscript. SP participated in the design of the study, data collection and interpretation. All authors read and approved the final manuscript.

## Pre-publication history

The pre-publication history for this paper can be accessed here:



## References

[B1] Foreyt J, Goodrick K (1995). The ultimate triumph of obesity. Lancet.

[B2] Nutrition and Physical Activity Task Force (1995).

[B3] Prescott-Clark P (1997). Health Survey for England 1995.

[B4] Kuczmarski RJ, Flegal KM, Campbell SM, Jonson CL (1994). Increasing prevalence of overweight among US adults: the National Health and Nutrition Examination Surveys 1960 to 1961. JAMA.

[B5] Kuskowa-Wolk A, Bergström R (1993). Trends in body mass index and prevalence of obesity in Swedish women 1980–89. J Epidemiol Community Health.

[B6] Seidell JC (1997). Time trends in obesity; an epidemiological perspective. Horm Metab Res.

[B7] Mabat (2000). Obesity Survey in Israel.

[B8] SIGN Obesity In Scotland: Integrating Prevention with Weight Management A National Clinical Guideline Recommended for Use in Scotland by the Scottish Intercollegiate Guidelines Network.

[B9] National Task Force on the Prevention and Treatment of Obesity (1996). Long term pharmacotherapy in the management of obesity. JAMA.

[B10] Pasternak S, Feigenbaum A, Sarid M, Dicker D Primary care physicians' attitude and practice using weight loss drugs. Abstract presented at the NAASO conference, Quebec City, Canada.

[B11] Bandura A (1997). Self-Efficacy The Exercise of Control.

[B12] Puska P, Nissinen A, Salonen JT, Toumilehto J (1983). Ten years of the North Karelia project: Results with community-based prevention of coronary heart disease. Scand J Soc Med.

[B13] Grimley DJO, Prochaska, Brinthaupt TM, Lipka RP  (1994). The transtheoretical model of change. Changing the Self: Philosophies, Techniques, and Experiences.

[B14] Prochaska JO, Johnson S, Shumaker SH, Schron E, Okene JK (2000). The transtheoretical model of change. The Handbook of Health Behavior Change.

[B15] Prochaska JO, Velicer WF (2000). The transtheoretical model of health behavior change. American Journal of Health Promotion.

[B16] Pintrich PR, DeGroot EV (1990). Motivational and self regulated learning components of classroom academic performance. J Educational Psychol.

[B17] Lent RW, Brown SD, Larkin KC (1986). Self efficacy in the prediction of academic performance and perceived career options. J Counseling Psychol.

[B18] Lent RW, Lopez FG, Bieschke KJ, Socall DW (1991). Mathematics self efficacy: Sources of relations to science-based career choice. J Counseling Psychol.

[B19] Bandura A (1986). Social Foundations of Thought and Action: A Social Cognitive Theory.

[B20] Denzin NK, Lincoln YS (1994). Handbook of Qualitative Research.

[B21] Glazer B, Strauss AL (1967). The Discovery of the Grounded Theory: Strategies for Qualitative Research.

[B22] Zeldin AL, Pajares F (2000). Against the odds: Self-efficacy beliefs of women in mathematical, scientific, and technological careers. Am Educational Res J.

[B23] Pajares F, Schunk DH, Riding R, Rayner S (1999). Self beliefs and school success: Self-efficacy, self-concept, and school achievement. International Perspectives on Individual Differences.

[B24] Pajares F, Schunk DH, Aronson J, Cardova D (1999). Self-efficacy, self-concept and academic achievement. Psychology of Education: Personal and Interpersonal Forces.

[B25] Katz S (2001). Appraisal of self-efficacy in regulating audience adaptation writing activities as a result of differential reflection and skill training. Proceedings of the American Educational Research Association Conference: New Orleans.

[B26] Tuckman BW, Sexton TL (1991). The effect of teacher encouragement on student self-efficacy and motivation for self regulated performance. J Social Behavior and Personality.

